# A Spironolactone-Based Prototype of an Innovative Biomedical Patch for Wound Dressing Applications

**DOI:** 10.3390/ijms25179608

**Published:** 2024-09-05

**Authors:** Giovanna Aquino, Gianluca Viscusi, Massimo Christian D’Alterio, Verdiana Covelli, Giuliana Gorrasi, Claudio Pellecchia, Paola Rizzo, Anna Maria D’Ursi, Giacomo Pepe, Chiara Amante, Pasquale Del Gaudio, Manuela Rodriquez

**Affiliations:** 1 Department of Pharmacy, University of Salerno, Via Giovanni Paolo II, 132, 84084 Fisciano, SA, Italy; gaquino@unisa.it (G.A.); dursi@unisa.it (A.M.D.); gipepe@unisa.it (G.P.); camante@unisa.it (C.A.); pdelgaudio@unisa.it (P.D.G.); 2 Department of Industrial Engineering, University of Salerno, Via Giovanni Paolo II, 132, 84084 Fisciano, SA, Italy; gviscusi@unisa.it (G.V.); ggorrasi@unisa.it (G.G.); 3 Department of Chemical Sciences, Università degli Studi di Napoli Federico II, Via Cintia, 80126 Napoli, NA, Italy; massimochristian.dalterio@unina.it; 4 Department of Pharmacy, University of Naples “Federico II” Via Domenico Montesano, 49, 80131 Napoli, NA, Italy; verdiana.covelli@unina.it; 5 Department of Chemistry, University of Salerno, Via Giovanni Paolo II, 132, 84084 Fisciano, SA, Italy; cpellecchia@unisa.it (C.P.); prizzo@unisa.it (P.R.)

**Keywords:** biomedical dressing, wound healing, electrospun fibers, drug release, *Ganoderma lucidum*

## Abstract

The electrospinning process is an effective technique for creating micro- and nanofibers from synthetic and natural polymers, with significant potential for biomedical applications and drug delivery systems due to their high drug-loading capacity, large surface area, and tunable release times. Poly(L-lactic acid) (PLLA) stands out for its excellent thermo-mechanical properties, biodegradability, and bioabsorbability. Electrospun PLLA nanofibrous structures have been extensively investigated as wound dressings, sutures, drug delivery carriers, and tissue engineering scaffolds. This study aims to create and characterize electrospun PLLA membranes loaded with spironolactone (SP), mimicking active compounds of *Ganoderma lucidum* (GL), to develop a biodegradable patch for topical wound-healing applications. GL, a medicinal mushroom, enhances dermal wound healing with its bioactive compounds, such as polysaccharides and ganoderic acids. Focusing on GL extracts—obtained through green extraction methods—and innovative drug delivery, we created new fibers for wound-healing potential applications. To integrate complex mixtures of bioactive compounds into the fibers, we developed a prototype using a single pure substance representing the extract mixture. This painstaking work presents the results of the fabricating, wetting, moisture properties, material resilience, and full characterization of the product, providing a robust rationale for the fabrication of fibers imbued with more complex extracts.

## 1. Introduction

The electrospinning process represents an excellent and versatile technique for producing micro- and nanofibers from various polymers, both synthetic and natural [1,2]. Electrospun fibers hold great promise for technological and biomedical applications, particularly as drug delivery systems, due to their high drug-loading capacity, large surface area, mechanical strength, porosity, and ability to deliver multiple therapeutic agents simultaneously [3,4,5]. In addition, biodegradable electrospun fibers can be used to fine-tune the release time of drugs. Poly(L-lactic acid) (PLLA) is gaining recognition as a leading biomaterial due to its exceptional biocompatibility, processability, and excellent thermo-mechanical properties, coupled with biodegradability and bioabsorbability [6].

By adjusting the solution characteristics and controlling the methods of electrospinning, the physical, morphological, biological, and mechanical properties of PLLA-based nanofibrous structures can be precisely controlled. While PLLA is generally considered safe, concerns have arisen from the biomedical use of tin, a cytotoxic agent, as a catalyst in its commercial production. To address this issue, the study switched from commercial tin-based PLLA to an in-house and bio-friendly zinc-based PLLA, which is widely recognized in the literature as non-toxic [7]. The applications of electrospun nanofibrous structures based on PLLA have been extensively investigated as artificial blood vessels, sutures, wound dressings, drug delivery carriers, and tissue engineering scaffolds [8]. 

Wound healing is a complex, multifaceted biological process involving several biological stages, including hemostasis, resolution of inflammation, cell proliferation, and tissue remodeling. An ideal wound dressing should protect the wound from bacterial infection, reduce inflammation, and promote cell proliferation to facilitate tissue reconstruction. Bioactive extracts, obtained from natural products, are well known for their beneficial effects on human health (e.g., anti-inflammatory, antioxidant, wound-healing, and antibacterial properties) and are widely used for cosmeceutical and nutraceutical purposes [9,10,11,12]. *Ganoderma lucidum* (GL) is a well-known medicinal mushroom used in traditional Chinese medicine due to its pharmacological properties, and its extracts are commonly recommended for their beneficial health effects, including anti-inflammatory, antioxidant, and wound-healing properties [13]. Although GL extracts have been shown to enhance dermal wound healing due to their rich content of polysaccharides, β-glucan, and ganoderic acids, there is limited research on the use of electrospun fibers loaded with GL extracts for wound-healing applications [14,15].

Given our intense interest in both GL extracts and innovative drug delivery, we embarked on the creation of new fibers with potential applications for the wound healing process [16]. To develop a robust system that could ensure a sustained- or controlled-release dosage of the complexity of active ingredients contained in GL extracts, we sought a pure bioactive molecule whose structure was representative of the lanostane-type triterpenoids (ganoderic acids)—the main class of active compounds with multiple pharmacological activities. The carbon skeleton of spironolactone (SP) well mimics the structure of ganoderic acids. 

Here, we report the manufacture and full characterization of electrospun membranes based on PLLA, loaded with SP, in order to obtain a biodegradable dressing as a prototype for the topical delivery of a class of molecules selected for the wound healing process [17].

The results outline a solid protocol for the manufacturing of dressings with PLLA fiber electrospun in the presence of complex natural extracts.

## 2. Results and Discussion

Spironolactone was selected as a pure bioactive molecule that is structurally mimetic with lanostane-type triterpenoids, the main class of active GL compounds (ganoderic acids) with multiple pharmacological activities, such as healing and anti-inflammatory, antiviral, antioxidant, and antitumor properties. Fibers loaded with spironolactone were first successfully fabricated and characterized to select a robust model for further loading with the much more precious active extracts. 

### 2.1. Optimization and Fabrication of Electrospun Membranes

The electrospinning conditions were first optimized to produce defect-free fibrous membranes. By adopting optimal conditions, fibers of pure PLLA and PLLA fibers loaded with spironolactone were successfully obtained. 

**Figure 1:**
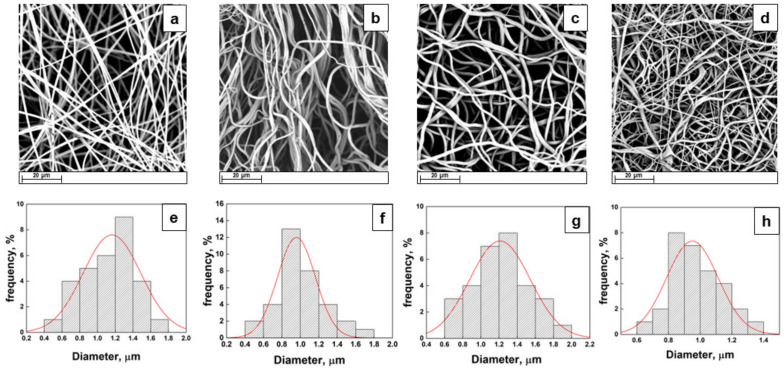
SEM images and diameter distributions of PLLA (**a**,**e**), PLLA + 2.5% SP (**b**,**f**), PLLA + 5% SP (**c**,**g**), and PLLA + 10% SP (**d**,**h**).

The morphology, thickness, and distribution of the active ingredient within the electrospun fiber scaffolds were imaged via scanning electron microscopy to ensure consistency in the fiber morphology among the electrospun samples of each polymer, as differences in the fiber diameter, density, or alignment may affect the release results. Representative scanning electron microscope (SEM) photographs of neat PLLA electrospun fibers and their relative fiber diameter distributions are shown in Figure 1.

The neat PLLA fibers appeared to possess a defect-free, randomly oriented fibrous structure with a mean diameter of 1.16 ± 0.36 µm. The resulting structure exhibited no nanometric morphology, but for the proposed application regarding drug delivery, the smooth and bead-less nanofiber structure has a more significant effect than the fiber diameter [18]. The presence of spironolactone did not noticeably affect the fiber morphology in terms of the mean dimension. The distributions reported in Figure 1 confirm that the mean diameter changes from 1.16 µm for neat PLLA and up to 0.95 ± 0.20 µm, 0.95 ± 0.16 µm, and 1.21 ± 0.33 µm for PLLA + 2.5% SP, PLLA + 5% SP, and PLLA + 10% SP, respectively. Probably, at low concentrations of spironolactone, its presence contributed to an increase in the conductivity of the solution, which is known to affect the whipping motion of the electrospinning jet, thus leading to thinner fibers. Moreover, at higher concentrations, such as 10% wt, the increase in viscosity led to a slight increase in the mean diameter [19]. Additionally, since no agglomerates are visible, it could be stated that the spironolactone is homogeneously distributed inside the polymeric fibers. No phase separation occurred during the electrospinning process, suggesting a good dispersion of the functional drug.

### 2.2. Morphology and Surface Analysis

**Figure 2:**
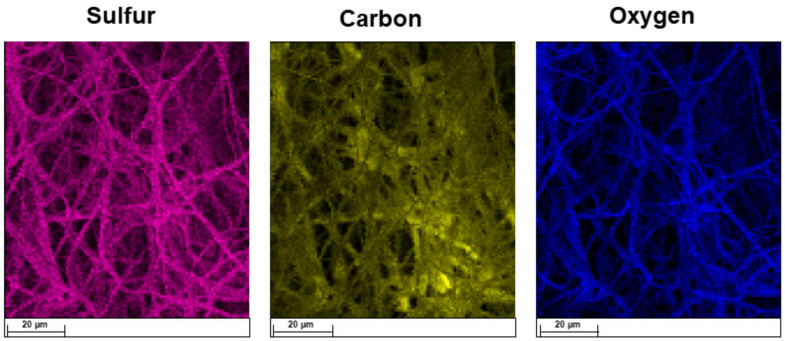
EDX elemental mapping of PLLA + 2.5% SP.

To confirm the distribution of SP inside the polymeric structure, energy dispersive X-ray (EDX) analysis was carried out, and the elemental maps are reported in Figure 2 [20]. By way of example, just maps of the PLLA + 2.5% SP were reported by highlighting some specific elements such as carbon, sulfur, and oxygen.

**Figure 3:**
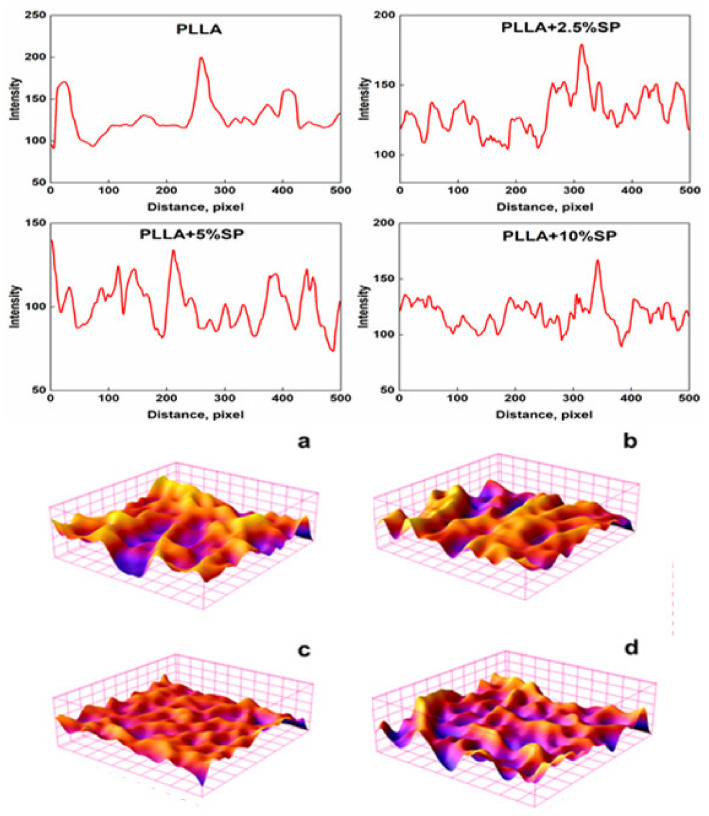
Top: profile plots of electrospun systems. Bottom: 3D surface plots of (**a**) PLLA, (**b**) PLLA + 2.5% SP, (**c**) PLLA + 5% SP, and (**d**) PLLA + 10% SP.

The investigation of SEM micrographs even allowed us to obtain the profile plots reported as a gray value (pixel intensity) vs. pixel distance (Figure 3).

In the three-dimensional surface plots, the topology clearly differs from each other in height. The regions with very low pixel intensity values are seen as blue, while the regions with high intensity appear to be red (Figure 3). In addition to the incorporation of SP, the arrangements of fiber can also affect the fiber surface roughness value. The surface roughness average (Ra) parameters were evaluated, and the Ra values obtained were 83, 120, 100, and 92 for PLLA, PLLA + 2.5% SP, PLLA + 5% SP, and PLLA + 10% SP. On the other hand, the RMS values calculated were 119, 144, 135, and 128 for PLLA, PLLA + 2.5% SP, PLLA + 5% SP, and PLLA + 10% SP, confirming the trend of the Ra parameters. The increase in surface roughness of the electrospun membrane is a crucial point since it is known that a rougher surface is important, as it can control the adhesion and differentiation of cells [21].

### 2.3. Thermal Properties

**Figure 4:**
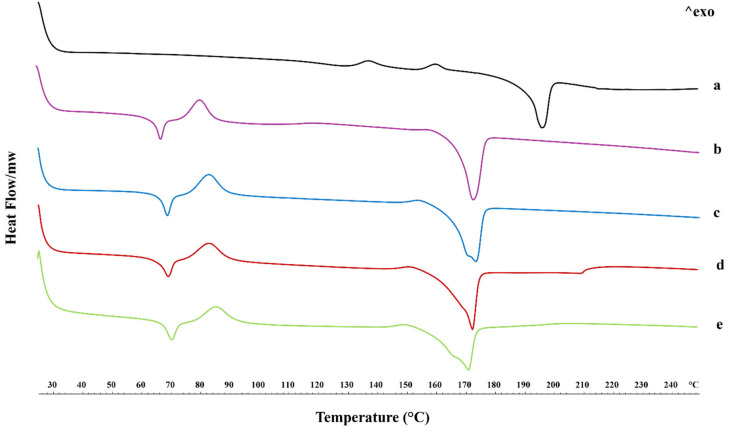
Differential scanning calorimetry (DSC) thermograms of spironolactone raw material (a), electrospun PLLA fibers (b), PLLA + 2.5% SP (c), PLLA + 5% SP (d), and PLLA + 10% SP (e).

To highlight the changes in the physical state of raw spironolactone, PLLA fibers, and spironolactone-loaded PLLA fibers, a differential scanning calorimetry (DSC) investigation was carried out, and the thermograms are reported in Figure 4. Focusing on the spironolactone thermal profile in Figure 4a, it is possible to observe two small exothermic peaks (in the middle of the thermogram) that could be related to the polymorphic nature of spironolactone (148 and 160 °C), followed by a sharp endothermic peak at 196 °C, representing the melting phenomenon [22,23].

The thermal behavior of electrospun PLLA fibers (Figure 4b) is characterized by an endothermic–exothermic sequence of peaks, such as the glass transition (Tg) and cold crystallization (Tcc) of polymer in a low range of temperatures (66 °C and 80 °C). In comparison, the melting peak (Tm) appeared at 176 °C [24,25].

The interesting endothermic–exothermic sequence is widely explained in the literature for electrospun PLLA because the solution conductivity affects the electrical force that may act on a fluid jet, causing the preferential orientation of the polymer chains [26].

Moving attention to the thermographs of the PLLA-SP-loaded fibers at different drug concentrations (i.e., 2.5, 5, and 10% *w/v*) (Figure 4c–e) in comparison to the drug-free PLLA fibers, the glass transition and the cold crystallization phenomena appeared shifted toward higher temperatures, with a widening of exothermic peak (described by the temperature range “ΔT” in Table 1) probably due to the presence of SP that delayed the polymer chain re-organization.

Similar to cold crystallization, the melting peak of PLLA broadly underlines the presence of SP and, thus, the less organized structure of crystals in the fibers (Table 1).

We can hypothesize that the presence of the drug hinders the crystallization of the PLLA in the ordered α form, inducing the formation of the more disordered-less compact α’ form. This is clearly evident by the small exothermic peak at around 150 °C, which is commonly attributed to the disordered–ordered transition from the α’ to α form and to the broadening of the melting peaks at ~170 °C [27].

On the other hand, the melting peak of the SP was not observed, indicating the stability of the drug in the amorphous form [28,29].

Additionally, the degree of crystallinity of the polymer samples was also examined using wide-angle X-ray diffraction (WAXD) analysis. The WAXD analysis on the membranes also showed that the PLLA resulted in being prevalently amorphous (Figure S1).

### 2.4. Wettability and Liquid Retention

**Figure 5:**
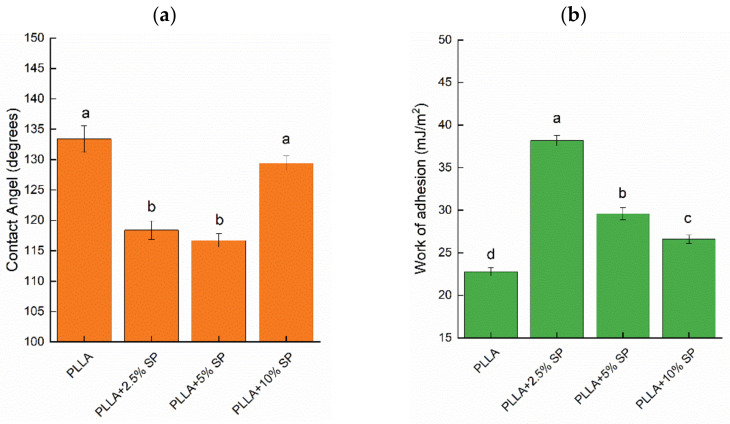
Water contact angle (**a**) and work of adhesion values (**b**). Different letters indicate that the mean values are significantly different (*p* ≤ 0.05).

In the design of effective adhesive patches, the wettability of the dressing is a key parameter, as it influences the adhesion, drug release, patient comfort, moisture management, and antimicrobial properties. Achieving the right balance of wettability improves the functionality and overall effectiveness of the plaster since the right level of wettability can balance the moisture effectively, preventing skin maceration and promoting a favorable healing environment. As known, the wettability of the material is also a key factor in biodegradation and biocompatibility [30]. To evaluate this important parameter, the hydrophobicity analysis on the fabricated electrospun membranes was carried out by the contact angle measurement. The values of the contact angles (CA) are reported in Figure 5a, while the values of the work of adhesion of the tested specimens are reported in Figure 5b.

In general, all the prepared membranes showed high CA values (CA ≥ 110°). They indicate that the water droplet does not easily spread on the surface because of the intrinsic hydrophobicity of the non-woven organization of the membrane, attributable to the microfiber morphology, which is responsible for the strong decrease in the wettability of these materials. The contact angles change, as shown in Figure 5, and are 133°, 118°, 116°, and 129° for PLLA, PLLA + 2.5% SP, PLLA + 5% SP, and PLLA + 10% SP, respectively. This might be attributed to the spaces created by the randomly distributed fibers, which kept the fibers apart and resulted in easier water droplet infiltration, thus showing a smaller water contact angle. Despite the increase in surface roughness after loading with 2.5% *w/w* and 5% *w/w* SP, as previously reported, the increased hydrophilicity could be due to the polar groups of spironolactone as well as the increased porosity, as shown by the SEM micrographs. With the addition of the drug, the decrease in CA proved the successful incorporation of SP into the electrospun fibers. The hydrophilic -OH group causes the lower water contact angle since π hydrogen bonds and surface defects could exist. This improvement in hydrophilicity will enhance tissue regeneration and increase the rate of membrane biodegradation, with these two drug-loading values representing the optimal balance for the best wettability of the patch.

For a higher amount of drug (10% wt), a further increase was recorded, which can be ascribed to the tighter structure, which can limit the water drop from spreading. This trend is even confirmed by the Neumann model, whose parameters are reported in Table 2. The surface free energy, γ_S_, and the β parameters did not show a monotonous trend with respect to the SP concentration. For low concentrations of the drug, the free surface energy reached the lowest value (21.92 mJ/m^2^) before increasing for higher amounts, and no substantial differences could be observed for PLLA + 5% SP and PLLA + 10% SP.

**Figure 6:**
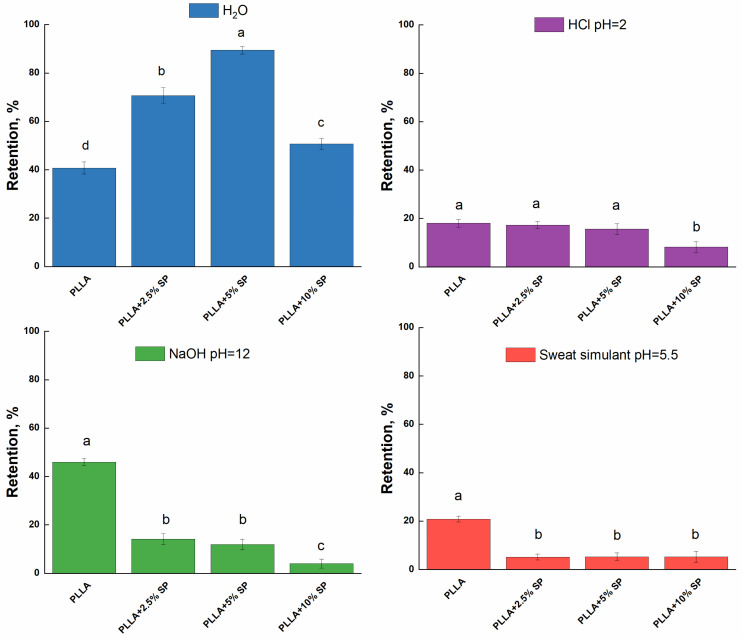
Liquid retention tests of the electrospun membranes. Different letters are significantly different according to Tukey’s honestly significant difference (HSD) at *p* < 0.05. The same letters indicate no significant difference between PLLA electrospun membranes.

Liquid retention of the electrospun dressings was performed by immersing a pre-weighed mass in liquid solutions with different pH conditions. pH values from 3 to 13, with intermediate values mimicking those of sweat, were considered. Figure 6 reports the liquid retention results obtained by testing four different liquid media.

The observed trend cannot be simply described since many overlapping factors affect the retained liquid amount, such as roughness, porosity, polar surface sites, and point of zero charge. Concerning the water retention test, the unloaded PLLA shows a retention degree of about 40%, which increases up to 90% for PLLA + 5% SP. The increase in the amount of water retained is consistent with the contact angle. The entry of a greater amount of water is therefore favored by the presence of the active ingredient. At a higher concentration (10%), the reduced surface roughness and the high amount of drug could potentially reduce the availability of polar sites as well as porosity, which may subsequently affect the reduction of the free volume. In the acidic medium, no significant differences were observed among the samples. The PLLA, PLLA + 2.5% SP, and the PLLA 5% SP could adsorb about 20% of the HCl-based medium, while a retention of about 8% was observed for PLLA + 10% SP. The protonation of surface groups due to the excess of H^+^ ions might limit the swelling of the polymeric system as well as the entry of water molecules. In basic conditions, the pristine polymer could adsorb about 46%. An evident decrease was recorded for loaded PLLA fibers (up to 4% for PLLA + 10% SP). The presence of -OH groups could enhance the formation of repulsive forces between the polymer chains, leading to a partial swelling of the polymeric network. The phenomenon could be limited by the presence of SP. Finally, for the sweat simulant, the behavior of membranes seems to follow the same trend observed for the acidic medium. It can be concluded that the presence of salts and ions, including sodium, chloride, and lactate, does not influence the swelling behavior or retention capacity of electrospun membranes.

### 2.5. Drug Release Profile

The in vitro cumulative release studies of the spironolactone from PLLA fibers were performed in the hydroalcoholic mixture (water/ethanol in a ratio of 90/10) to compare the release of spironolactone from the fibers loaded with different amounts of the drug (2.5, 5, and 10% *w/w*).

The amount of drug incorporated into the PLLA fibers has resulted in being perfectly in line with the predicted data, obtaining fibers with 2.5, 5, and 10% *v/v* of the drug based on the amount of polymer. These results can be ascribed to the chemical nature of the drug. In fact, during the electrospinning process, if the drug is compatible with the matrix/solvent system, its entrapment will result in being extremely efficient [31].

**Figure 7:**
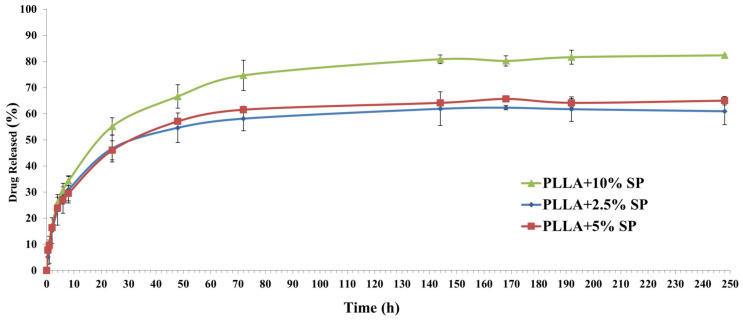
Spironolactone released from the PLLA electrospun fibers containing 10, 5, and 2.5% *w/w* of the drug.

Regarding the release profile, as reported in Figure 7, all fibers exhibited a similar curve typical of the electrospinning matrix: a burst release followed by a prolonged release [32].

Specifically, all fibers release about 50% of spironolactone after 24 h, followed by a more sustained release until 240 h. It is possible to notice that the fiber that contains more spironolactone results in a higher amount of the drug being released, probably due to the different interactions between the polymers and drug since the dynamic of drug release from the electrospinning matrix is controlled by its structure.

## 3. Materials and Methods

The polymeric scaffold is an isotactic Poly-L-Lactic Acid (PLLA) with a tailored molecular mass (M_n_ = 27.5 kDa, M_w_/M_n_ = 1.8) and a melting temperature of 174.7 °C. The PLLA was purposely synthesized with a zinc-based catalyst bearing an imidazole [1,5-a]pyrid-3-yl)phenolate ligand [33]. The spironolactone was purchased from Sigma Aldrich (Milan, Italy).

### 3.1. Preparation of Electrospun Membranes

The electrospun membranes were prepared by dissolving the polymer in a solvent mixture of DCM/DMF (80:20 *v/v*) at 12% *w/w*. Spironolactone was added to the PLLA solution at different drug-to-polymer ratios (2.5%, 5%, 10% *w/w*) and mixed for 4 h at 40 °C (350 rpm) using a temperature-controlled stirring plate to obtain a homogenous solution. The electrospun membranes were obtained by using a one-nozzle setup with a stainless-steel capillary (Ø_int_ = 0.8 mm) (EM-CAX-Ime electrospinning). After that, the polymeric solution was fed to a 5 mL syringe pump. The electrospinning parameters were optimized to produce nanofibrous mats without bead formation. Finally, since spironolactone was selected as being structurally mimetic of the chemical molecular scaffold of the class most represented in the GL extract, a preliminary test concerning the fabrication of GL extract-loaded PLLA electrospun membrane was carried out through the optimization of the maximum loadable percentage of extract.

### 3.2. Physico-Chemical Characterization

Scanning electron microscopy (SEM) was carried out using a Phenom ProX microscope working in the high-vacuum mode and coupled with an Energy Dispersive X-ray Analysis (EDX) probe.

Differential scanning calorimetry (DSC, Mettler Toledo DSC 822e module controlled by Mettler STARe software, v.16.30, Columbus, OH, USA) was used to determine the thermal characteristics of the spironolactone, PLLA fibers, and spironolactone-loaded fibers. A total of 2–3 mg of the sample was weighed with a microbalance (MTS Mettler Toledo, Columbus, OH, USA) before being placed in a perforated aluminum pan (40 µL). The temperature range was 25 to 250 °C at a rate of 10 °C/min, and the characteristic peaks were recorded. All DSC analyses were performed in 150 mL/min under a nitrogen atmosphere. An empty pan was used as a reference. Data were collected with the Mettler Toledo STARe software (v.16.30) and analyzed using thermal analysis instruments to characterize the materials’ thermal events and degradation profiles.

Water contact angle measurements were performed using a high-resolution camera. Droplets of distilled water (100 μL) were dispensed onto the fibrous mat. The contact angle was determined using the Drop Analysis plugin (Image J software, v.1.53t). Five tests were carried out for each sample.

SEM analysis was conducted to investigate the morphology of the fibers. Before the analysis, the samples were covered with a thin film of gold by sputtering. The images were acquired by a Phenom ProX microscope, working in the high-vacuum mode coupled with an EDX probe. The diameters of the fibers were evaluated by analyzing the digital images. The fiber diameters were obtained from the recorded photographs and are expressed as the mean ± standard deviation. The plot profiles were obtained from the SEM images through the Plot Profile plugin of the Fiji software v.1.54f. Image processing analysis allows the display of a two-dimensional graph of the intensities of pixels by selecting an area of roughly 1000 μm^2^. The x-axis represents the distance (pixel), and the y-axis is the pixel intensity. 

Finally, the surface roughness parameters were evaluated through mathematical equations. The arithmetic average of the absolute values of the profile height deviations from the mean line (R_a_) and the root mean square average of the profile height deviations from the mean line, recorded within the evaluation length (L) (RMS), were evaluated through Equations (1) and (2):

(1) $$R_{a}=\frac{1}{L}\times \int_{0}^{L}\left|Z\left(x\right)\right|dx$$

(2) $$RMS={\left[\frac{1}{L}\times \int_{0}^{L}{Z\left(x\right)}^{2}dx\right]}^{\frac{1}{2}}$$

The liquid retention of the electrospun fabrics was performed by immersing a pre-weighed mass of a sample (previously dried for 8 h under vacuum at 50 °C and weighed (M_0_)) in 25 mL of different liquid solutions (pure water, HCl solution (pH = 3), NaOH solution (pH = 13), and sweat simulant (pH = 5.5)). The sweat simulant was prepared according to EN 1811 by dissolving NaCl (10.8 g), lactic acid (1.2 g), and urea (1.3 g) in 1 L of distillate water. The pH was adjusted to 5.5 by dropping the NaOH solution. Liquid retention tests were performed at 37 °C for 1 h. After that, the samples were wiped and re-weighed (M_eq_). The retention degree was evaluated according to Equation (3).

(3) $$Retention\left(\%\frac{g}{g(d.b.)}\right)=\frac{M_{0}-M_{eq}}{M_{0}}\times 100$$

Contact angle (CA) measurements were performed using a high-resolution camera. Droplets of liquids (100 μL) were dispensed on a 1 × 1 cm^2^ test sample at room temperature. The contact angle was determined using Drop Analysis software (Fiji software v.1.54f). Five contact angle measurements were recorded at different points for each sample, from which the average contact angle, the surface energy, and the work of adhesion were determined. The estimation of CA allowed to evaluate the total surface energy of a solid using the Neumann method [34] (Equation (4)):

(4) $$\ln\left[\gamma_{L}\times {\left(\frac{1+cos\theta }{2}\right)}^{2}\right]=-2\beta \times {\left(\gamma_{S}-\gamma_{L}\right)}^{2}+\ln\left(\gamma_{S}\right)$$

where γ_s_ is the surface free energy (mJ/m^2^); γ_L_ is the liquid free surface energy; θ is the contact angle; and β is a parameter related to the solid surface. The characteristic parameters of the probe liquids used to evaluate the contact angles are summarized in Table 3 [34,35].

The thermodynamic work of adhesion W_A_ is the energy required to separate a unit area of interface and can be described by the Young–Dupré equation [36] (Equation (5)):

(5) $$W_{A}=\gamma_{L}\left(1+\cos\theta \right)$$

### 3.3. Spironolactone Content

To evaluate the capability of the PLLA electrospun fibers to load the GL extract, the spironolactone content, chosen as a molecular scaffold mimetic, was calculated. The actual drug content within the prepared SP electrospun fibers, calculated as the ratio between the spironolactone detected and the total fiber weight, was determined. Pre-weighted fibers (circular discs of 1–3 mg) were dissolved in 2 mL of absolute ethanol. All the samples were vortexed for 5 min and centrifuged (5000 rpm for 10 min), and the supernatant obtained was filtered (filters of 0.2 µm) and analyzed by HPLC [37,38].

The Agilent 1100 Series instrument (Agilent 1100 Series HPLC, Agilent Technologies, Santa Clara, CA, USA), with a UV detector set at 238 nm, was used for the HPLC analysis. The separation was performed on a reversed-phase column (Synergi 4µ Fusion-RP 80A 250 mm × 4.6 mm, Phenomenex, Torrance, CA, USA), using acetonitrile and deionized water 0.1% trifluoroacetic acid (60:40) as a mobile phase, with an injection volume of 20 μL and a flow rate of 1 mL/min. The HPLC calibration curve was linear (R^2^ = 0.9998) in the 1.25–200 µg/mL concentration range.

### 3.4. Spironolactone Release

The spironolactone-loaded electrospun fibers (circular discs of 2–4 mg) were immersed in glass vials containing 6.5 mL of the hydroalcoholic mixture (water/ethanol in a ratio of 90/10) to maintain the sink conditions to compare the release of spironolactone from the fibers. The fibers exhibited good wettability and remained completely immersed without additional support throughout the experiment. The glass vials were maintained at 37 °C in an orbital shaker, setting the stirrer at 150 rpm. At appropriate intervals from 30 min to 250 h, 400 μL of the samples were withdrawn and replenished with an identical volume of fresh buffer. The drug release was determined by HPLC, as previously reported.

### 3.5. Statistical Analysis

The statistical significance of the obtained data was assessed by performing a one-way ANOVA test. Tukey’s post hoc method was conducted to assess the pairwise comparison (*p* < 0.05). The statistical comparisons were obtained by means of the Statistix 8.1 software (Analytical Software, Miller Landing Rd, Tallahassee, FL, USA).

## 4. Conclusions

Spironolactone was chosen as a pure bioactive molecule that is structurally mimetic with lanostane-type triterpenoids, which are the main class of active ganoderic acids in *Ganoderma lucidum*, with multiple pharmacological activities such as healing, anti-inflammatory, antiviral, antioxidant, and antitumor properties. To develop a robust model for drug delivery, fibers loaded with spironolactone were first successfully fabricated and characterized.

The electrospinning conditions were first optimized to produce defect-free fibrous membranes. SEM was used to ensure a consistent fiber morphology, thickness, and distribution of the active ingredient. The SEM images showed defect-free, randomly oriented fibers with mean diameters that changed slightly with different spironolactone concentrations, and the elemental mapping confirmed the homogeneous distribution of spironolactone within the fibers. The surface roughness of the fibers was observed to increase with the drug loading. The optimal values, which were found to be beneficial for cell adhesion and differentiation, were identified as occurring when the SP was present at concentrations of 2.5 and 5%. The investigation of thermal properties indicates that the drug, in its amorphous state, is stable within the fibers.

A critical factor for the effectiveness of adhesive patches is wettability. Although all fibers showed high contact angles, these decreased slightly with the increasing SP concentration, presumably due to the increase in fiber porosity. Both the wettability and liquid retention tests revealed that PLLA fibers with 2.5% SP showed the optimal profile required to enhance adhesion, drug release, prevention of skin maceration, and the promotion of a favorable healing environment.

In conclusion, this study successfully developed and characterized spironolactone-loaded PLLA fibers using electrospinning. The fibers demonstrated an appropriate morphology, uniform drug distribution, enhanced surface roughness, and desirable thermal properties. The tunable hydrophobic nature and controlled drug release profile make these fibers promising candidates for drug delivery systems, particularly in wound-healing applications. The ability to tailor the properties by adjusting the bioactive compound concentrations allows for customization to meet specific therapeutic needs. The findings of the comprehensive process outline a solid protocol to be followed in the manufacturing of dressings, where PLLA fibers will be electrospun in the presence of highly complex natural extracts. This protocol will hopefully provide a solid foundation for future work in this area.

## Figures and Tables

**Table 1:** Characteristic thermal data of electrospun PLLA and SP-loaded PLLA fibers.

	Tg (°C)	Tcc (°C)	ΔTcc (°C)	Tm (°C)	ΔTm (°C)
PLLA	67.05	80.26	15.31	172.55	22.43
PLLA + 2.5% SP	68.71	82.77	19.90	173.05	26.67
PLLA + 5% SP	69.04	82.92	21.30	172.20	27.50
PLLA + 10% SP	70.57	85.60	22.34	171.14	36.32

**Table 2:** Parameters evaluated from the Neumann method where γs is the solid free surface energy, and β is a parameter related to the solid surface.

	β (m^4^/mJ^2^)	γ_S_ (mJ/m^2^)
PLLA	0.0031	49.65
PLLA + 2.5% SP	0.0061	21.92
PLLA + 5% SP	0.0049	54.34
PLLA + 10% SP	0.0048	56.06

**Table 3:** Characteristic parameters of water, glycerol, and ethylene glycol.

Title 1	γ_L_ (mJ/m^2^)	γ^d^_L_ (mJ/m^2^)	γ^p^_L_ (mJ/m^2^)
Water	72.8	21.8	51
Glycerol	63.4	33.4	30
Ethylene Glycol	48	29	19

## Data Availability

The datasets used and/or analyzed during the current study are available from the corresponding author upon reasonable request.

## Supplementary Materials

The supporting information can be downloaded at: https://www.mdpi.com/article/10.3390/ijms25179608/s1.
