# Supplementary material for: A Spironolactone-Based Prototype of an Innovative Biomedical Patch for Wound Dressing Applications
Source: Int J Mol Sci. 2024 Sep 5;25(17):9608. doi: 10.3390/ijms25179608 (PMC11395607; doi:10.3390/ijms25179608)
Supplement: Supplementary file 1 [file ijms-25-09608-s001.zip › ijms-3156768-supplementary.pdf]

Supplementary Materials

A Spironolactone-Based Prototype of an Innovative Biomedical Patch for Wound Dressing Applications

Giovanna Aquino [1], Gianluca Viscusi [2], Massimo Christian D'Alterio [3], Verdiana Covelli [4], Giuliana Gorrasi [2], Claudio Pellecchia [5], Paola Rizzo [5], Anna Maria D'Ursi [1], Giacomo Pepe [1], Chiara Amante [1], Pasquale Del Gaudio [1] and Manuela Rodriquez [4,*]

[1] Department of Pharmacy, University of Salerno, Via Giovanni Paolo II, 132, 84084 Fisciano, SA, Italy; gaquino@unisa.it (G.A.); dursi@unisa.it (A.M.D.); gipepe@unisa.it (G.P.); camante@unisa.it (C.A.); pdelgaudio@unisa.it (P.D.G.)
[2] Department of Industrial Engineering, University of Salerno, Via Giovanni Paolo II, 132, 84084 Fisciano, SA, Italy; gviscusi@unisa.it (G.V.); ggorrasi@unisa.it (G.G.)
[3] Department of Chemical Sciences, Università degli Studi di Napoli Federico II, Via Cintia, 80126 Napoli, NA, Italy; massimochristian.dalterio@unina.it
[4] Department of Pharmacy, University of Naples "Federico II" Via Domenico Montesano, 49, 80131 Napoli, NA, Italy; verdiana.covelli@unina.it
[5] Department of Chemistry, University of Salerno, Via Giovanni Paolo II, 132, 84084 Fisciano, SA, Italy; cpellecchia@unisa.it (C.P.); prizzo@unisa.it (P.R.)
* Correspondence: manuela.rodriquez@unina.it

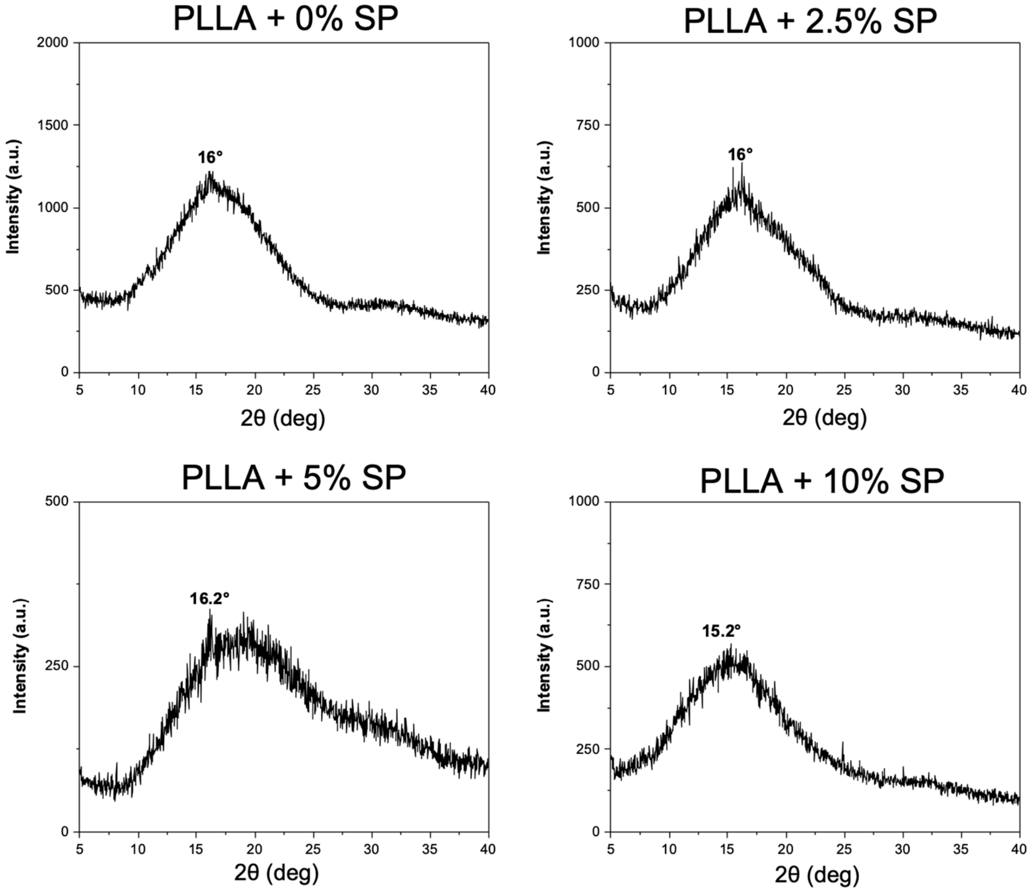

Figure S1. The WAXD profiles of PLLA, PLLA+2.5% SP, PLLA+5% SP and PLLA+10% SP showed that PLLA polymer samples resulted to be prevalently amorphous.
